# Delayed Recovery of Skeletal Muscle Mass following Hindlimb Immobilization in mTOR Heterozygous Mice

**DOI:** 10.1371/journal.pone.0038910

**Published:** 2012-06-22

**Authors:** Susan M. Lang, Abid A. Kazi, Ly Hong-Brown, Charles H. Lang

**Affiliations:** Department of Cellular and Molecular Physiology, Pennsylvania State University College of Medicine, Hershey, Pennsylvania, United States of America; University of Rome La Sapienza, Italy

## Abstract

The present study addressed the hypothesis that reducing mTOR, as seen in mTOR heterozygous (+/−) mice, would exaggerate the changes in protein synthesis and degradation observed during hindlimb immobilization as well as impair normal muscle regrowth during the recovery period. Atrophy was produced by unilateral hindlimb immobilization and data compared to the contralateral gastrocnemius. In wild-type (WT) mice, the gradual loss of muscle mass plateaued by day 7. This response was associated with a reduction in basal protein synthesis and development of leucine resistance. Proteasome activity was consistently elevated, but atrogin-1 and MuRF1 mRNAs were only transiently increased returning to basal values by day 7. When assessed 7 days after immobilization, the decreased muscle mass and protein synthesis and increased proteasome activity did not differ between WT and mTOR^+/−^ mice. Moreover, the muscle inflammatory cytokine response did not differ between groups. After 10 days of recovery, WT mice showed no decrement in muscle mass, and this accretion resulted from a sustained increase in protein synthesis and a normalization of proteasome activity. In contrast, mTOR^+/−^ mice failed to fully replete muscle mass at this time, a defect caused by the lack of a compensatory increase in protein synthesis. The delayed muscle regrowth of the previously immobilized muscle in the mTOR^+/−^ mice was associated with a decreased raptor•4EBP1 and increased raptor•Deptor binding. Slowed regrowth was also associated with a sustained inflammatory response (e.g., increased TNFα and CD45 mRNA) during the recovery period and a failure of IGF-I to increase as in WT mice. These data suggest mTOR is relatively more important in regulating the accretion of muscle mass during recovery than the loss of muscle during the atrophy phase, and that protein synthesis is more sensitive than degradation to the reduction in mTOR during muscle regrowth.

## Introduction

The plasticity of skeletal muscle is evidenced by the hypertrophy seen with strength training and, conversely, the atrophy seen with disuse [1], [2]. This atrophic response can be generalized when produced by extended periods of bed rest or localized when a single limb is immobilized [3]–[9]. This muscle wasting when sustained and severe can be a determinant of morbidity and mortality in chronic catabolic states, and results from an imbalance between rates of protein synthesis and breakdown. Previous studies have demonstrated that these metabolic processes change over time and are in part dependent on the etiology of the atrophic stimuli [8], [10]–[13]. While the net catabolic response in muscle leads to the release of amino acids supporting select immune and metabolic responses, the sustained loss of muscle mass decreases strength and impairs ambulation [3], [14], [15]. Conversely, muscle reloading during the recovery phase results in the gradual accretion of mass and the ability to generate force [3], [16]. As restoration of muscle mass is necessary to re-establish normal function, a greater understanding of mechanisms by which muscle is lost and regained is of clinical importance.

The mammalian target of rapamycin (mTOR) is the catalytic component of two multiprotein complexes, referred to as mTORC1 and mTORC2, which collectively regulate diverse cellular functions including protein balance [17]. The impairment in muscle protein synthesis produced by disuse is associated with both a reduction in the phosphorylation of downstream targets of mTORC1 such as eukaryotic initiation factor (eIF) 4E-binding protein-1 (4E-BP1) and ribosomal protein S6 kinase-1 (S6K1) [6],[18]–[21] as well as S473-phosphorylation of Akt which is a recognized substrate for mTORC2 [18], [20]. Additionally, inhibition of mTOR signaling enhances muscle protein degradation via activation of the ubiquitin proteasome system [22]. In general, mTOR senses and integrates inputs from nutrients, energy status and growth factors, as evidenced by the severe atrophy observed in mice with muscle-specific ablation of mTOR [23]. However, whether alterations in mTOR are causally related to the reduced muscle protein synthesis and increase in protein degradation produced by immobilization in adult animals is unclear. As mice with whole-body mTOR deletion (e.g., knockout [KO]) are embryonically lethal [24], [25] and mice with muscle-specific mTOR KO have a severe muscle pathology and die prematurely [23], we addressed this question using mice which are heterozygous (+/−) for mTOR with an ∼50% reduction in total mTOR protein in skeletal muscle and other tissues [26]. Using these haploinsufficient mice and their wild-type (WT) littermates, we addressed the hypothesis that reduction of mTOR would exaggerate the changes in both protein synthesis and degradation observed during a sustained period of hindlimb immobilization as well as impair normal muscle regrowth during the recovery period.

## Materials and Methods

### Animal protocols

All mice were housed under specific pathogen-free conditions and controlled environmental conditions (12∶12 light:dark). Mice were provided Teklad Global 2019 (Harlan Teklad, Boston, MA) and water *ad libitum*. All breeding and experimental protocols were approved by the Institutional Animal Care and Use Committee of The Pennsylvania State University College of Medicine and adhered to the National Institutes of Health (NIH) guidelines for the use of experimental animals.

Experimental mice were either mTOR heterozygous (mTOR^+/−^) or WT male littermates (12–16 wks, with a body weight of 26–28 g). The generation and phenotypic characterization of these mice has been previously described [26]. Briefly, Western blot analysis of gastrocnemius, heart and liver from mTOR^+/−^ mice indicated a ∼50–60% reduction in the amount of total mTOR protein [26]. Whole-body mTOR heterozygous mice were used instead of a muscle-specific mTOR KO because various catabolic insults decreased mTOR phosphorylation by ∼50% under *in vivo* conditions [17], and there are no naturally occurring physiological or catabolic conditions characterized by the complete loss of mTOR protein in skeletal muscle. Moreover, mice with muscle-specific knockout of mTOR exhibit a serve wasting and permature death [23]. Therefore, while the use of whole-body mTOR h3eterozygous mice somewhat limits data interpretation, we believe this animal model represents a more physiological experimental paradigm.

Mice were anesthetized with isoflurane (2–3% in O_2_+1.5% maintenance) for hindlimb immobilization [12]. Briefly, hair on the left lower hindlimb was removed with clippers, the skin swabbed with 70% alcohol, and the hindlimb wrapped with a single layer of surgical tape. A small amount of generic superglue was applied to the tape and a 1.5 ml plastic microfuge tube without the lid was placed over the leg, maintaining the foot in a plantar-flexed position to induce maximal atrophy of the gastrocnemius. The bottom of the tube was also removed so as to prevent condensation inside the tube. The weight of the tube was ∼500 mg (∼2% of body weight) and did not appear to limit animal mobility. The contralateral leg was not immobilized and served as the internal control. The contralateral leg does not undergo hypertrophy (see Table 1), and the validity of this unilateral hindlimb immobilization has been previously reported for other animal models [27], [28]. Each mouse received 1 ml of warmed (37°C) sterile 0.9% saline for resuscitation. After casting, mice were housed individually for various periods of time with food and water *ad libitum*. Food was placed in both the overhead bin and within the cage to permit easy access. A second study was performed in which the hindlimb was immobilized for 7 days and then the cast removed so muscle recovery could be assessed at various time points. In some mice, body composition was monitored non-invasively in conscious animals using a ^1^H-NMR analyzer (Bruker LF90 Proton-NMR Minispec: Bruker Optics, Woodlands, TX) for rapid measurement of total body lean and fat mass [26]. In a separate study, WT mice were used at either after: a) 5 days of immobilization or b) after 7 days of immobilization and 10 days of recovery. Mice in this study were fasted for 4 h and then administered an oral gavage of either leucine (1.35 g/kg body wt) or an equal volume of saline, and skeletal muscle was excised 30 min thereafter. The dose and timing of leucine administration were based on prior studies demonstrating maximal stimulation of muscle protein synthesis as well as mTOR signaling [26].

**Table 1:** Comparison of unilateral and bilateral hindlimb immobilization (“casting”) on body weight, food intake and muscle weight.

	Control (naive)		Unilateral casting		Bilateral Casting	
	Pre	Post	Pre	Post	Pre	Post
Body weight, *grams*	25.6±0.4	26.1±0.4*	25.1±0.3	25.7±0.6*	24.9±0.5	24.7±0.6
Food Consumption, g/day	4.2±0.3	4.3±0.3	4.1±0.3	4.2±0.4	4.0±0.4	3.6±0.5*
	**Control (naive)**		**Unilateral Casting**		**Bilateral Casting**	
	**Control**	**Control**	**Control**	**Casted**	**Control**	**Casted**
Muscle Wt, *mg*	111±5a	110±6a	109±5a	88±4b	85±4b	83±4b

Values are means ± SEM; n = 8 mice per group. Hindlimb was immobilized for 5 days. “Pre” food consumption was determined daily for 3 days prior to hindlimb immobilization and the values averaged. For body weight and food consumption, ^+^ *P*<0.05, compared to respective pre-value for each group, determined using paired *t*-test. For muscle weight, values with a different superscript are statistical different from each other as determined by ANOVA and Student-Newman-Keuls (*P*<0.05).

### In vivo protein synthesis

The *in vivo* rate of protein synthesis in the gastrocnemius (hereafter referred to as muscle) was determined in the control and immobilized limb at various time points after immobilization (“casting”). Protein synthesis was determined using the flooding-dose technique, as described [8], [12], [26], [29]. Mice were injected intraperitoneal with [^3^H]-L-phenylalanine (150 mM, 30 µCi/ml; 1 ml/100 g body wt), and blood was collected 15 min later from the abdominal aorta. The plasma phenylalanine concentration and radioactivity was measured by HPLC analysis of supernatant from TCA extracts of plasma. Thereafter, muscles were rapidly excised. A portion of muscle was freeze-clamped, stored at −70°C, and processed as previously described [8], [29], [30]. The rate of protein synthesis was calculated by dividing the amount of radioactivity incorporated into protein by the plasma phenylalanine-specific radioactivity. The advantages and disadvantages of this method have been reviewed [31]. In addition, samples of fresh muscle were homogenized for Western blot and analysis of selected proteins and another piece of tissue used for qRT-PCR, as described below.

### Western blotting and immunoprecipitation

Fresh skeletal muscle was homogenized (Kinematic Polytron; Brinkmann, Westbury, NY) in ice-cold homogenization buffer consisting of (in mmol/L): 20 HEPES (pH 7.4), 2 EGTA, 50 sodium fluoride, 100 potassium chloride, 0.2 EDTA, 50 β-glycerophosphate, 1 DTT, 0.1 phenylmethane-sulphonylfluoride, 1 benzamidine, and 0.5 sodium vanadate. The protein concentration was quantified using the Bio-Rad protein assay kit (Bio-Rad, Hercules, CA) and equal amounts of total protein per sample were subjected to standard SDS-PAGE. The blots were incubated with primary antibodies to total and phosphorylated proteins (Cell Signaling, Beverly, MA, unless otherwise noted), including total and phosphorylated 4E-BP1 (Thr 37/46; Bethyl Laboratories, Montgomery, TX), total and phosphorylated S6 (S240/244), total and phosphorylated Akt (S473), total and phosphorylated PRAS40 (proline-rich Akt substrate 40; Biosource, Camarillo, CA), total S6K1, total raptor (regulatory-associated protein of TOR), and total Deptor (DEP-domain partner of mTOR; Millipore, Billerica, MA). Tubulin and eIF4E were, on occasion, used as loading controls. Blots were developed with enhanced chemiluminescence Western blotting reagents (Supersignal Pico, Pierce Chemical, Rockford, IL). Dried blots were exposed to x-ray film to achieve a signal within the linear range and film was then scanned (Microtek ScanMaker IV; Cerritos,CA) and quantified using Scion Image 3b software (Scion Corporation, Frederick, MD). Additionally, eIF4E was immunoprecipitated from aliquots of supernatants using an anti-eIF4E monoclonal antibody (Drs. Jefferson and Kimball; Hershey, PA). Antibody-antigen complexes were collected using magnetic beads subjected to SDS-PAGE. The 4E-BP1•eIF4E and eIF4G•eIF4E complexes were then quantified, as described above.

To maintain potential protein-protein interactions within mTORC1, fresh muscle was homogenized in CHAPS buffer (pH 7.5) composed of the following (in mM): 40 HEPES, 120 NaCl, 1 EDTA, 10 pyrophsophate, 10 β-glycerophosphate, 50 NaF, 1.5 sodium vanadate, 0.3% CHAPS, and one protease inhibitor cocktail tablet. The homgenate was clarified by centrifugation, and an aliquot of the supernatant was combined with anti-raptor antibody and immune complexes were isolated with goat anti-rabbit BioMag IgG (PerSeptive Diagnostics, Boston, MA) beads. The beads were collected, washed with CHAPS buffer, precipitated by centrifugation, and subjected to SDS-PAGE and quantitated as described above.

### *RNA* extraction and real-time quantitative *PCR*

Total RNA was extracted using Tri-reagent (Molecular Research Center, Inc., Cincinnati, OH) and RNeasy mini kit (Qiagen, Valencia, CA) following manufacturers’ protocols. Skeletal muscle was homogenized in tri-reagent followed by chloroform extraction according to the manufacturer’s instruction. An equal volume of 70% ethanol was added to the aqueous phase and the mixture was loaded on a Qiagen mini spin column. The Qiagen mini kit protocol was followed from this step onwards including the on-column DNase I treatment to remove residual DNA contamination. RNA was eluted from the column with RNase-free water and an aliquot was used for quantitation (NanoDrop 2000, Thermo Fisher Scientific, Waltham, MA). Quality of the RNA was analyzed on a 1% agarose gel. Total RNA (1 µg) was reversed transcribed using superscript III reverse transcriptase (Invitrogen, Carlsbad, CA) following manufacturer’s instruction. Real-time quantitative PCR was performed using 25 ng of cDNA in a StepOnePlus system using TaqMan gene expression assays (Applied Biosystems, Foster City, CA) for: Atrogin 1 (F-box protein 32; Mm00499523_m1), muscle RING-finger 1 (MuRF1; Mm01185221_m1), interleukin (IL)-6 (Mm00446190_m1), tumor necrosis factor (TNF)-α (Mm00443258_m1), CD45 (Mm01293575_m1), insulin-like growth factor-I (IGF-I; Mm00439560_m; all IGF-I transcripts) and glyceraldehyde-3-phosphate dehydrogenase (GAPDH; Mm99999915_g1). The comparative quantitation method 2^-ΔΔCt^ was used in presenting gene expression of target genes in reference to the endogenous control [32].

### Proteasome activity assay

Gastrocnemius was homogenized in 4 volumes of ice-cold 50 mM Tris (pH 7.5) containing 1 mM EDTA, 150 mM NaCl, 5 mM MgCl_2_, 50 µM ATP, and 0.5 mM DTT followed by centrifugation at 12,000×g for 30 min at 4°C. The proteasome enzymatic activity was measured by using a proteasome 20 S assay kit (Enzo Life Sciences, Farmingdale, NY) following the manufacturer's instructions. Briefly, determination of the chymotrypsin-like protease activity of the 20 S proteasome in samples was initiated by addition of a fluorogenic peptidyl substrate succinyl-Leu-Leu-Val-Tyr-7-amido-4-methylcoumarin (Suc-LLVY-AMC) to wells of a 96-well plate containing 50 µg of homogenized muscle sample. This substrate was cleaved by the proteasome activity and the subsequently released free AMC was then detected by a fluorometer (SpectraMax M5; Molecular Devices Corporation, Sunnyvale, CA) using an excitation wavelength of 360 nm and emission wavelength of 460 nm. The fluorescence signal was monitored before and at 10-min for 2 hours at 37°C. The change in fluorescence signal was normalized to the amount of protein used in the assay and all assays were in the linear range. Each sample/substrate combination was measured both in the presence and in the absence of MG132, a highly specific 20 S proteasome inhibitor (Boston Biochem, Cambridge, MA) to account for any non-proteasomal degradation of the substrate. These fluorescence units were then subtracted from each measurement. Results are expressed as µmol/mg protein/min or presented as percent of time-matched control value.

### Plasma IGF-I and insulin concentrations

The plasma concentrations of total IGF-I and insulin were measured using commercial ELISA kits (Diagnostic Systems Laboratory, Webster, TX and Linco Research, St. Louis, MO, respectively).

### Statistics

Data for each condition are summarized as means ± standard error of the mean (SEM) where the number of mice per treatment is indicated in the legend to the figure or table. Statistical evaluation of the data was performed using ANOVA with post-hoc Student-Neuman-Keuls test when the interaction was significant. To compare changes in the immobilized muscle to the non-immobilized control muscle in the same mouse, a 2-tailed paired *t*-test was performed. Differences between groups were considered significant at *P*<0.05. GraphPad Prism version 5.0 (GraphPad software, La Jolla, CA) was used for statistical analysis.

## Results

### Model characterization

**Figure 1:**
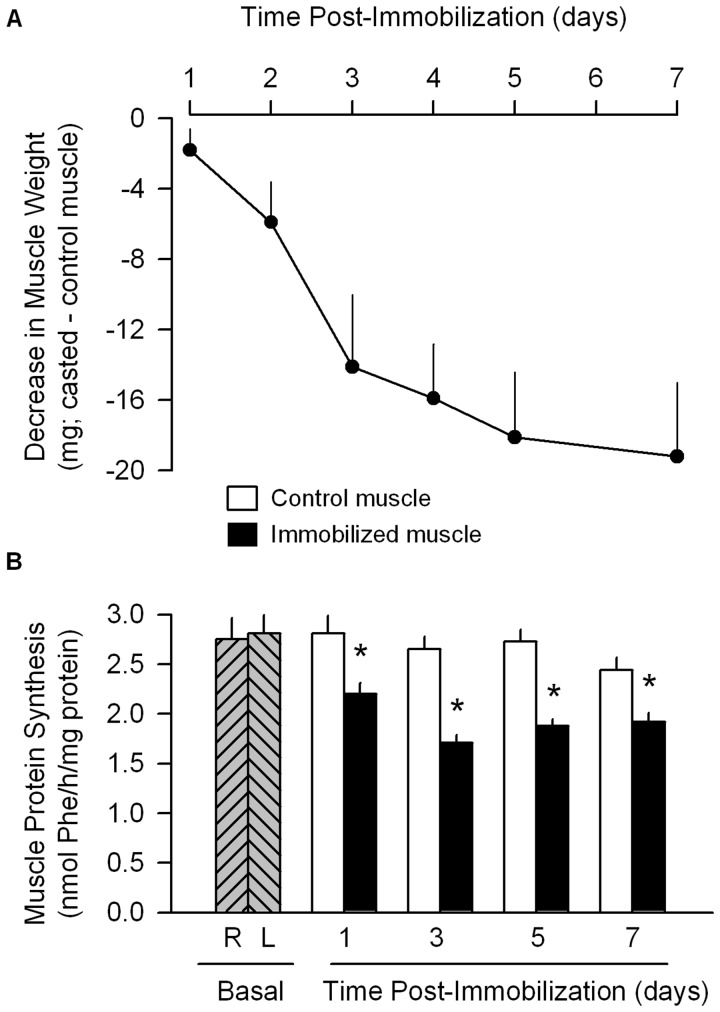
Temporal progression of changes in muscle mass and protein synthesis after unilateral hindlimb immobilization. (A) Decrement in mass of gastrocnemius at various times after immobilization, compared to contralateral non-casted muscle from the same mouse. All values from day 2–7 are statistically (*P*<0.05) decreased. Values are means ± SEM; n = 7−9 per time point. (B) *In vivo*-determined protein synthesis of gastrocnemius from immobilized and control (e.g., non-immobilized) muscle at various times after casting. First two cross-hatched bars, represent protein synthesis of the right (R) and left (L) gastrocnemius from naive control mice. *P<0.05, compared to contralateral non-casted control muscle of the same mouse. Values are means ± SEM; 7–9 mice per group.

An initial study was performed to determine whether unilateral or bilateral hindlimb immobilization was the preferable model for investigating disuse atrophy in mice. Naive control mice gained approximately 0.5 g over the 5 day observation period. Body weight and food consumption of mice where only one hindlimb was immobilized was not different from that of naive control mice (Table 1). In contrast, mice which had both hindlimbs immobilized failed to gain weight and consumed ∼10% less food than control animals. The reduced food intake by bilaterally casted mice was not due to their inability to obtain food because food was provided within the animal’s cage. The same magnitude of muscle mass loss was detected in the immobilized muscle from animals in which either one or two hindlimbs were casted. The weight of the uncasted muscle in the unilateral casted group did not differ from that of muscle of naive control mice. Finally, the *in vivo*-determined rate of protein synthesis in control muscle from unilateral casted mice did not differ from the synthetic rate of muscles from naive control mice (Figure 1B). Collectively, these data suggest bilateral immobilization is more stressful to mice than immobilization of a single hindlimb and the gastrocnemius from the uncasted limb does not undergo either a compensatory hypertrophy or a change in protein synthesis. Hence, all subsequent studies were performed using unilateral immobilization.

Figure 1A illustrates the progressive decrease in weight of the gastrocnemius from the immobilized limb over the 7-day course of study. This atrophic response appeared to plateau between 5–7 days after casting. *In vivo*-determined protein synthesis was decreased as early at 24 h after immobilization and this reduction was sustained for at least 7 days (Figure 1B). Protein synthesis was reduced 25–30% in the immobilized muscle, compared to the contralateral muscle, during this time period.

**Figure 2:**
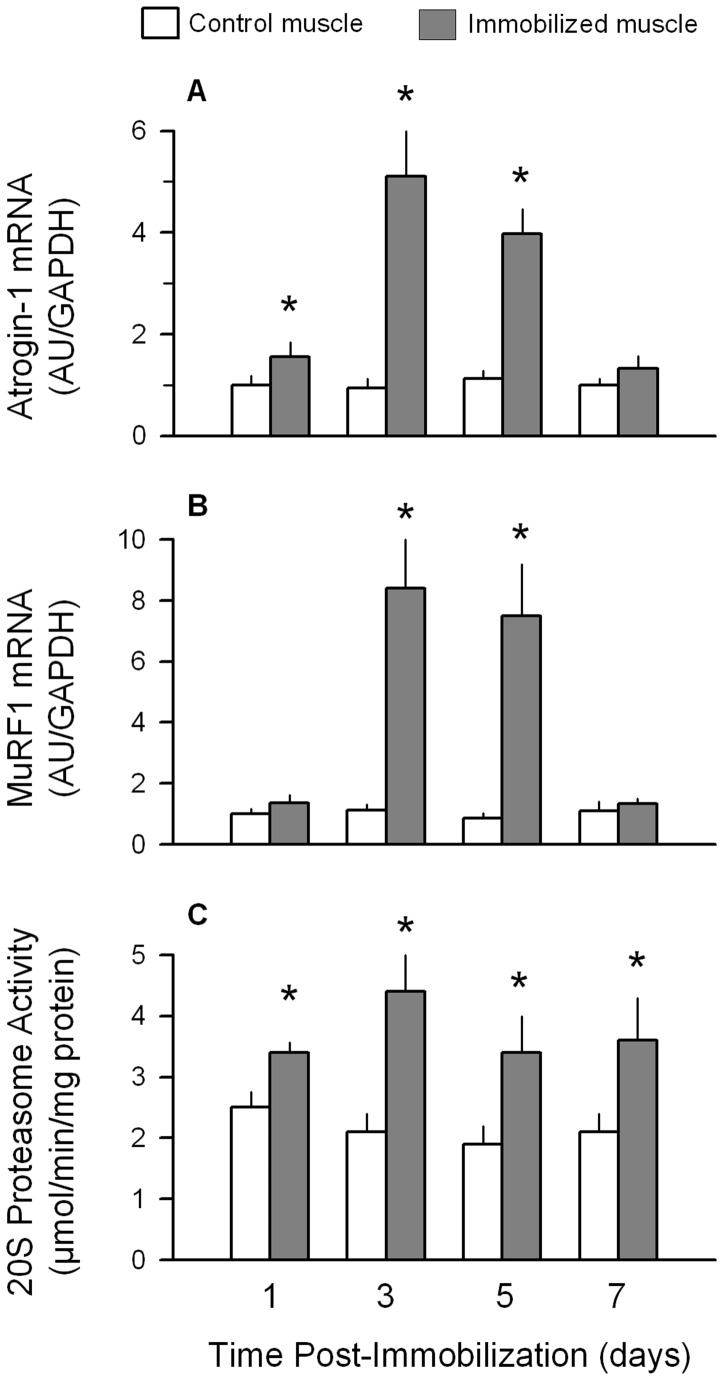
Temporal progression of changes in atrogin-1 and MuRF1 mRNA content and proteasome activity after unilateral hindlimb immobilization. (A) Quantitation of mRNA content of gastrocnemius for atrogin-1 at various times after unilateral hindlimb immobilization, compared to contralateral non-casted control muscle. (B) Quantitation of mRNA for MuRF1 in gastrocnemius at various times after immobilization. All mRNA data were normalized to GAPDH and the WT control value was set at 1.0 arbitrary units (AU/GAPDH). (C) 20 S proteasome activity determined in immobilized and control muscle. *P<0.05, compared to contralateral non-casted control muscle of the same mouse. Values are means ± SEM; 7–9 mice per group.

Temporal changes in the other side of the protein balance equation, that is protein degradation, were also assessed. The muscle-specific E3 ligases atrogin-1 and MuRF1, collectively referred to as atrogenes, are increased in disuse atrophy as well as a number of diverse catabolic conditions [8], [10], [18], [26], [33]–[36]. The mRNA content for atrogin-1 was slightly, albeit statistically, increased (60%) 1 day after immobilization (Figure 2A), with the increase peaking between days 3–5, before returning to control values by day 7. The immobilization-induced increase in MuRF1 was comparable to that seen for atrogin-1, except levels were not increased at the 1 day time point (Figure 2B). In contrast to the relatively transient changes in the mRNA content for these two E3 ligases, proteasome activity was consistently increased (approximately 75%) from day 1 through day 7 of immobilization (Figure 2C).

**Figure 3:**
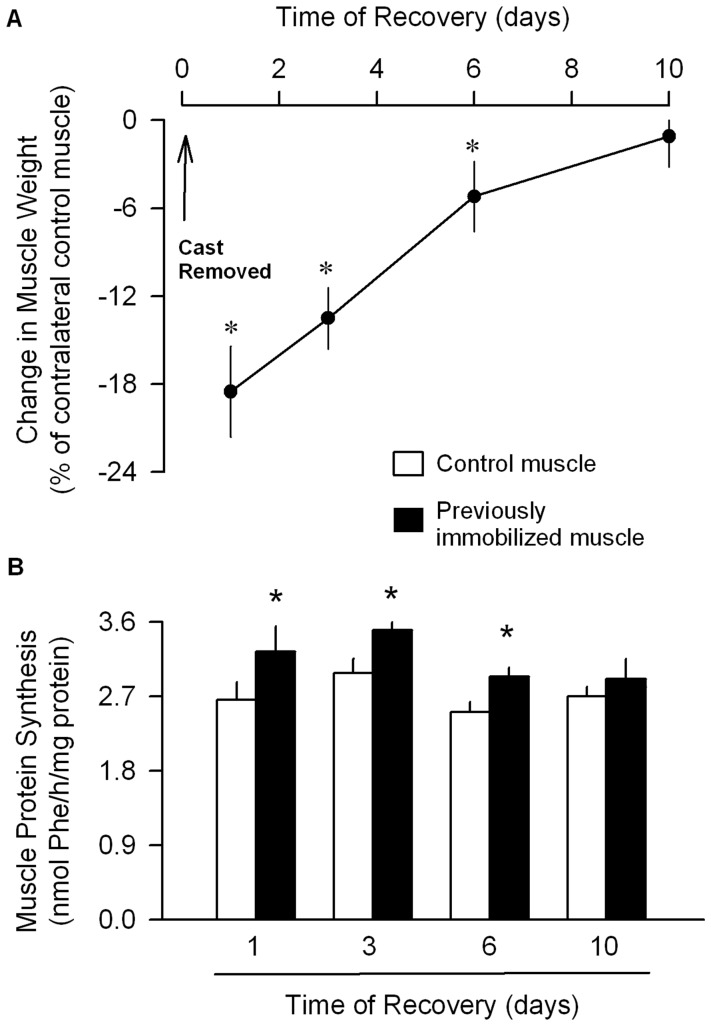
Temporal progression of changes in muscle mass and protein synthesis at various times after cast removal and muscle reloading. (A) Hindlimb immobilization was produced for 7 days, the cast removed, and the change in gastrocnemius mass determined at various times during the recovery period. Data are expressed as a change in muscle weight of the immobilized limb compared to that of the contralateral limb in the same mouse. (B) In vivo-determined protein synthesis of gastrocnemius from previously immobilized (7 days) and control muscle at various times after cast removal. For both graphs, *P<0.05, compared to contralateral non-casted control muscle from the same mouse. Values are means ±SEM; 7-9 mice per group.During recovery, there was no statistically significant difference between the previously immobilized muscle and the non-casted control muscle at any time point for either atrogin-1 or MuRF1 mRNA content (Figure 4A and 4B). In contrast, the proteasome activity in the previously immobilized muscle was increased 70% on day 1 of recovery, but was not different from control values at all other recovery time points.

We also examined the temporal progression for the recovery of muscle mass following cast removal and muscle reloading. The mass of the previously immobilized muscle gradually increased from day 1 through day 10 of the recovery period (Figure 3A). The weight of the previously immobilized muscle was reduced, compared to the contralateral non-casted muscle, from days 1–6. However, by day 10 of recovery, there was no difference in the weight of the two muscles. This recovery pattern was accompanied by an increased protein synthesis from days 1–6 which averaged ∼20% above control values (Figure 3B). However, protein synthesis did not differ between the previously immobilized and the non-casted control muscle at the 10-day time point.

**Figure 4:**
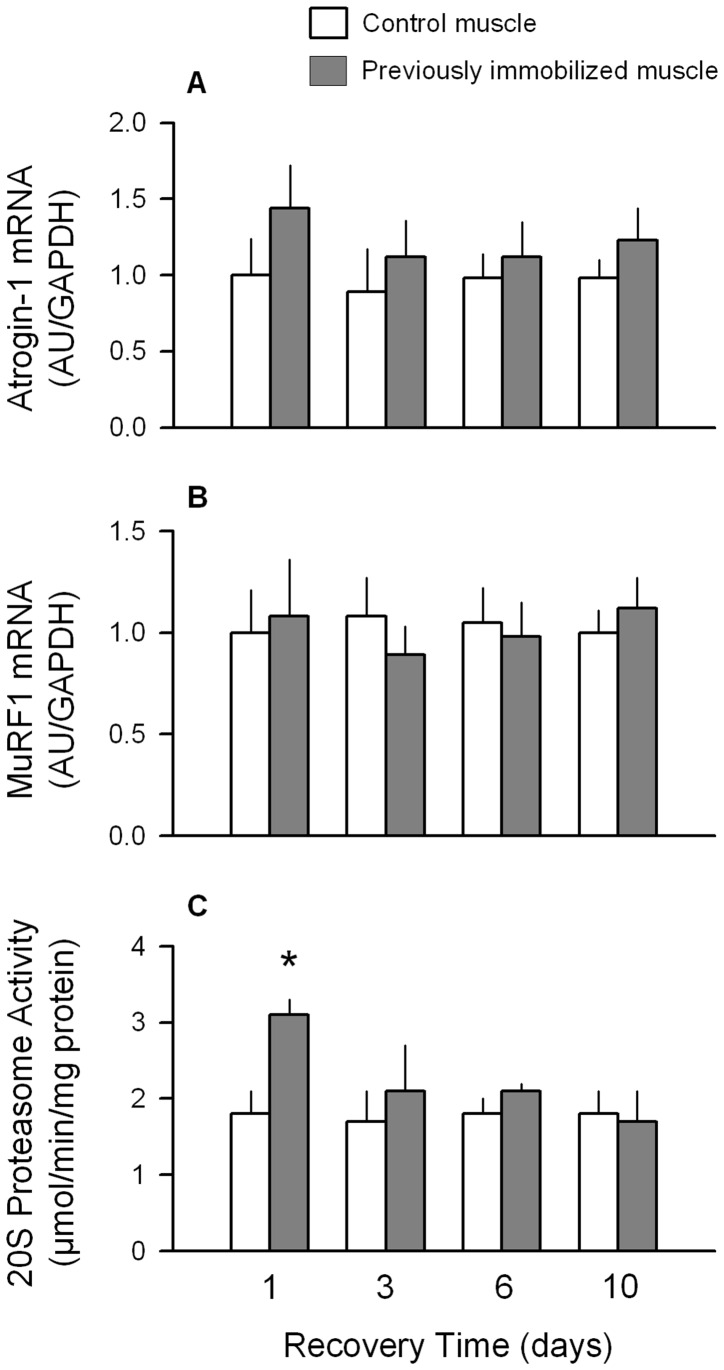
Temporal progression of changes in atrogin-1 and MuRF1 mRNA content and proteasome activity after cast removal and muscle reloading. (A–B) Quantitation of mRNA content of gastrocnemius for atrogin-1 and MuRF1, respectively, at various times during the recovery period. Hindlimb immobilization was produced for 7 days, the cast removed, and muscles excised at various time points thereafter. All data were normalized to GAPDH and the WT control value was set at 1.0 arbitrary units (AU/GAPDH). (C) 20 S proteasome activity determined in immobilized and control muscle after cast removal. *P<0.05, compared to contralateral non-casted control muscle for the same mouse. Values are means ± SEM; 7–9 mice per group.

During recovery, there was no statistically significant difference between the previously immobilized muscle and the non-casted control muscle at any time point for either atrogin-1 or MuRF1 mRNA content (Figure 4A and 4B). In contrast, the proteasome activity in the previously immobilized muscle was increased 70% on day 1 of recovery, but was not different from control values at all other recovery time points.

### Immobilization produces muscle leucine resistance

**Figure 5:**
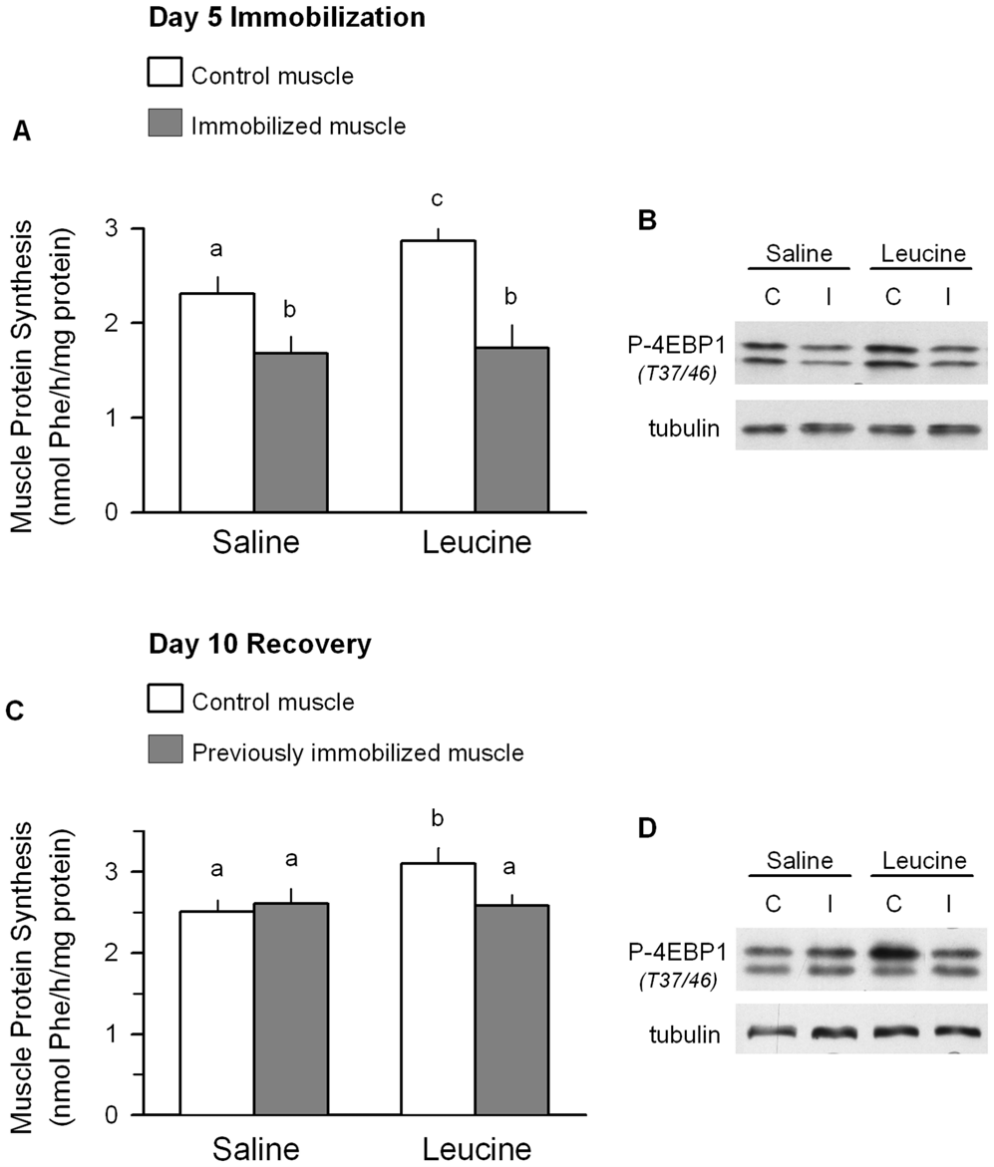
Immobilization-induced muscle leucine resistance. Unilateral hindlimb immobilization was produced for either 5 days (A–B) or for 7 days followed by a 10 day recovery period (C–D). (A and C) Protein synthesis was determined in both the immobilized and contralateral control muscle from 4 h fasted WT mice which were orally gavaged with either leucine or an equal volume of saline. Values with different superscript letters (a,b) are statistically significant (*P*<0.05). Values are means ± SEM; 7–9 mice per group. (B and D) Representative Western blot for Thr37/46-phosphorylated 4E-BP1 (top band  =  γ-isoform; bottom band  =  β isoform) in muscle from mice similarly treated. Blots are representative of n = 5−6 per group. Tubulin was used a loading control and demonstrated equal loading.

Next, unilateral hindlimb immobilization was produced for 5 days and then animals were orally gavaged with a maximally-stimulating dose of the branch-chain amino acid leucine or saline as a control. The 5-day time point was selected based on the previously presented data indicating atrophic muscle was characterized by a decrease in protein synthesis and an increase in proteasome activity and atrogene expression. Mice gavaged with saline showed the typical decrease in muscle protein synthesis in the immobilized muscle (Figure 5A). In mice orally administered leucine, the rate of protein synthesis in the control (i.e., non-immobilized) muscle was greater than that seen in the control muscle from the saline-treated group. However, leucine failed to increase muscle protein synthesis in the immobilized muscle. Immobilization- and leucine-induced changes in the extent of 4E-BP1 Thr37/46-phosphorylation were detected (Figure 5B) and were comparable to those seen for protein synthesis. The ability of leucine to stimulate protein synthesis and 4E-BP1 phosphorylation was still blunted in the previously immobilized muscle which was permitted to recover for 10 days (Figure 5C and 5D, respectively). As muscle was sampled at 30 min after leucine administration, leucine-induced change in atrogene mRNA or proteasome activity were detected (data not shown).

### Impact of hindlimb immobilization and muscle reloading on body composition of WT and mTOR+/− mice

**Figure 6:**
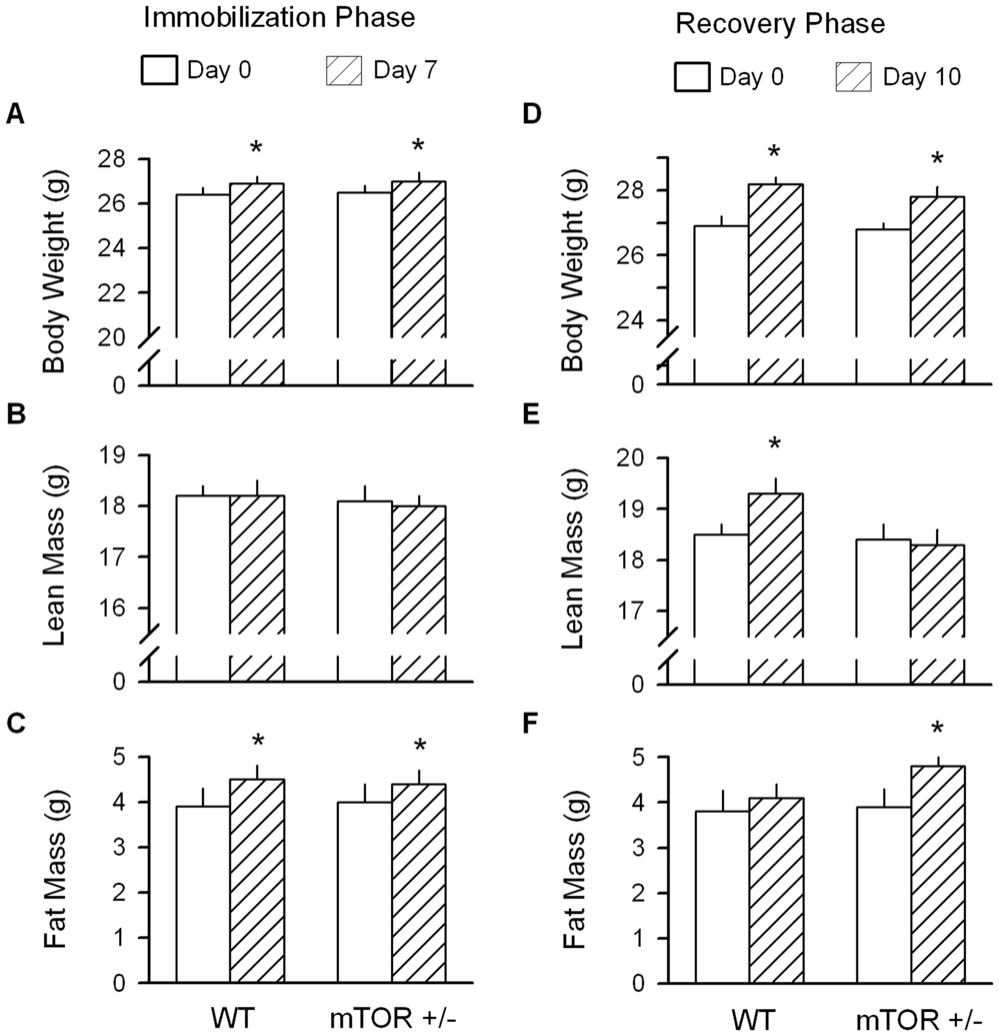
Effect of immobilization and reloading in wild-type (WT) and mTOR^+/−^ mice on body composition. (A–C) Quantitation of body composition determined in WT and mTOR^+/−^ mice immediately prior to and 7 days after unilateral hindlimb immobilization. (D–F) Quantitation of body composition determined in WT and mTOR^+/−^ mice on day 10 of muscle reloading which was preceded by 7 days of immobilization (e.g., day 0). For all bar graphs, values are means ± SEM; 7–9 mice per group. *P<0.05, compared to respective time-matched value from WT mice.

There was no difference in the body weight, lean mass or fat mass of WT and mTOR^+/−^ mice at the start of the experiment (i.e., basal condition) (Figure 6A, 6B and 6C, respectively). After 7 days of unilateral hindlimb immobilization, the increase in body weight was comparable for both WT and mTOR^+/−^ mice. The elevated body weight in both groups was primarily, if not exclusively, due to an increased fat mass.

Body weight, lean mass and fat mass did not differ between WT and mTOR^+/−^ mice at the time of cast removal (Figure 6D, 6E and 6F, respectively). After a 10-day recovery period, WT mice exhibited an increased body weight which could be solely accounted for by an increased lean mass, with no significant increased in fat mass. In contrast, while mTOR^+/−^ mice showed a comparable increase in body weight during the recovery period, this change was due to an increase in fat as opposed to lean mass.

### Role of mTOR in mediating protein balance during immobilization and recovery

**Figure 7:**
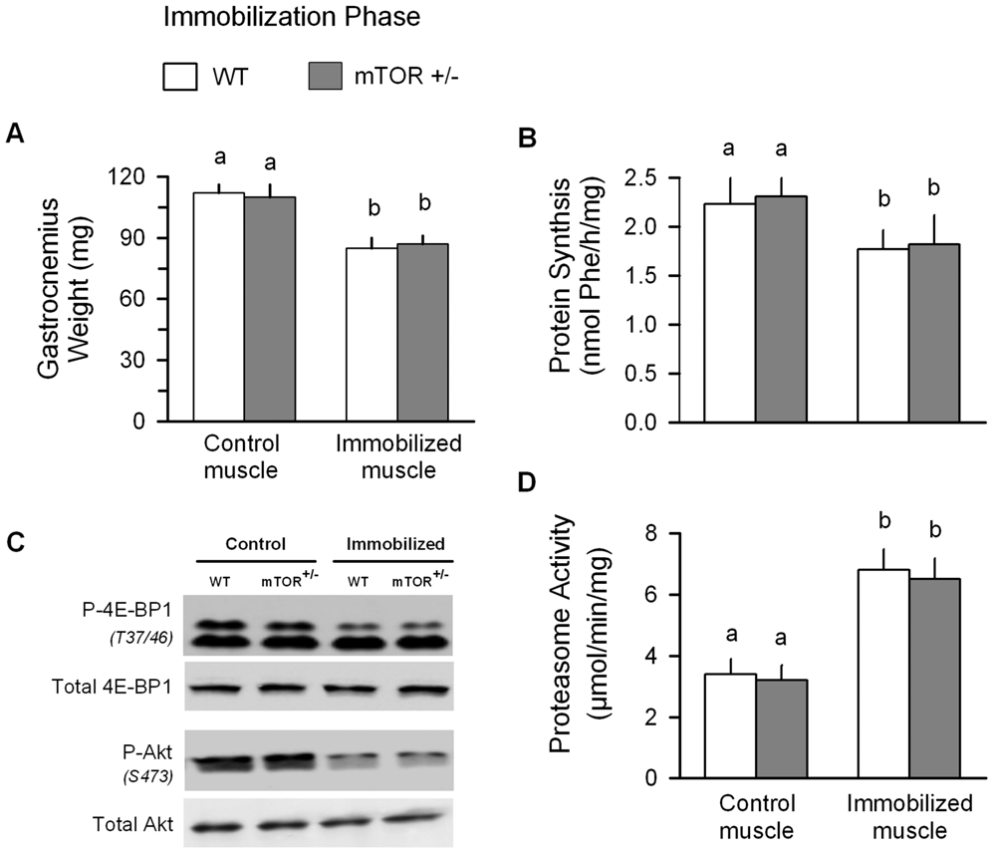
Protein metabolic effects of immobilization in wild-type (WT) and mTOR^+/−^ mice. All parameters were determined in control and immobilized muscle from WT and mTOR^+/−^ mice 7 days after unilateral hindlimb casting. Values with different superscript letters (a,b) are statistically significant (*P*<0.05). For all bar graphs (A, B and D), values are means ± SEM; 7–9 mice per group. (C) Western blot data are representative of n = 5 per group.

Hindlimb immobilization in WT mice produced the expected decrease in muscle mass which was accompanied by a decreased protein synthesis (Figure 7A and 7B, respectively). mTORC1 and mTORC2 signaling was assessed by determining the phosphorylation of 4E-BP1 (T37/46) and Akt (S473), respectively [37]. In WT mice, immobilization decreased the phosphorylation of both 4E-BP1 and Akt, without significantly reducing the total amount of either protein (Figure 7C). Conversely, proteasome activity of immobilized muscle from WT mice was increased 2-fold, compared to the control muscle from the same animals (Figure 7D). Finally, these immobilization-induced changes in protein metabolism did not differ between WT and mTOR^+/−^ mice.

**Figure 8:**
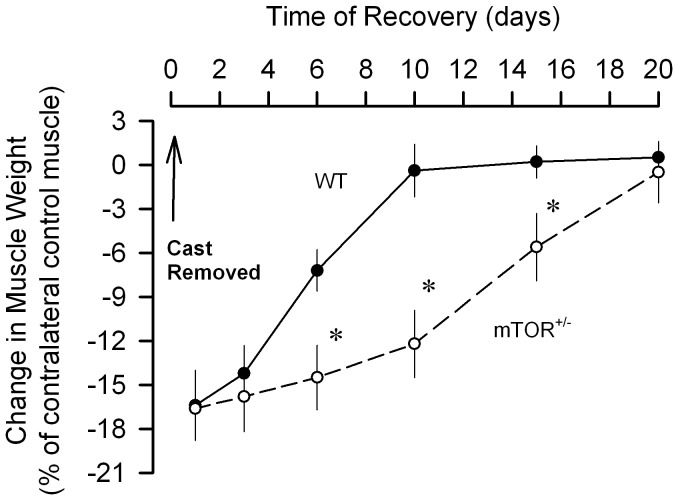
Temporal progression of regrowth in previously immobilized muscle in wild-type (WT) and mTOR^+/−^ mice. Mice had one hindlimb casted for a period of 7 days, the cast removed, and muscle weight assessed at various time points during recovery. Values are means ± SEM; 5–6 mice per group. *P<0.05, compared to time-matched value from WT mice.

To determine whether the temporal progression of muscle recovery after immobilization differed between WT and mTOR^+/−^ mice, a separate experiment was performed in which both groups of mice were subjected to hindlimb immobilization for 7 days, the cast removed and the muscles examined at various times after reloading. Figure 8 illustrates the weight of previously immobilized muscle from WT mice had recovered to that of the control muscle after 10 days of reloading. In contrast, the weight of previously immobilized muscle from mTOR^+/−^ mice was statistically lower at days 6, 10 and 15 days of recovery, with muscle weight not achieving control values until approximately day 20 following cast removal. Because the difference between WT and mTOR^+/−^ mice was maximal after 10 days of reloading, additional studies characterizing skeletal muscle protein balance were performed at this time point.

**Figure 9:**
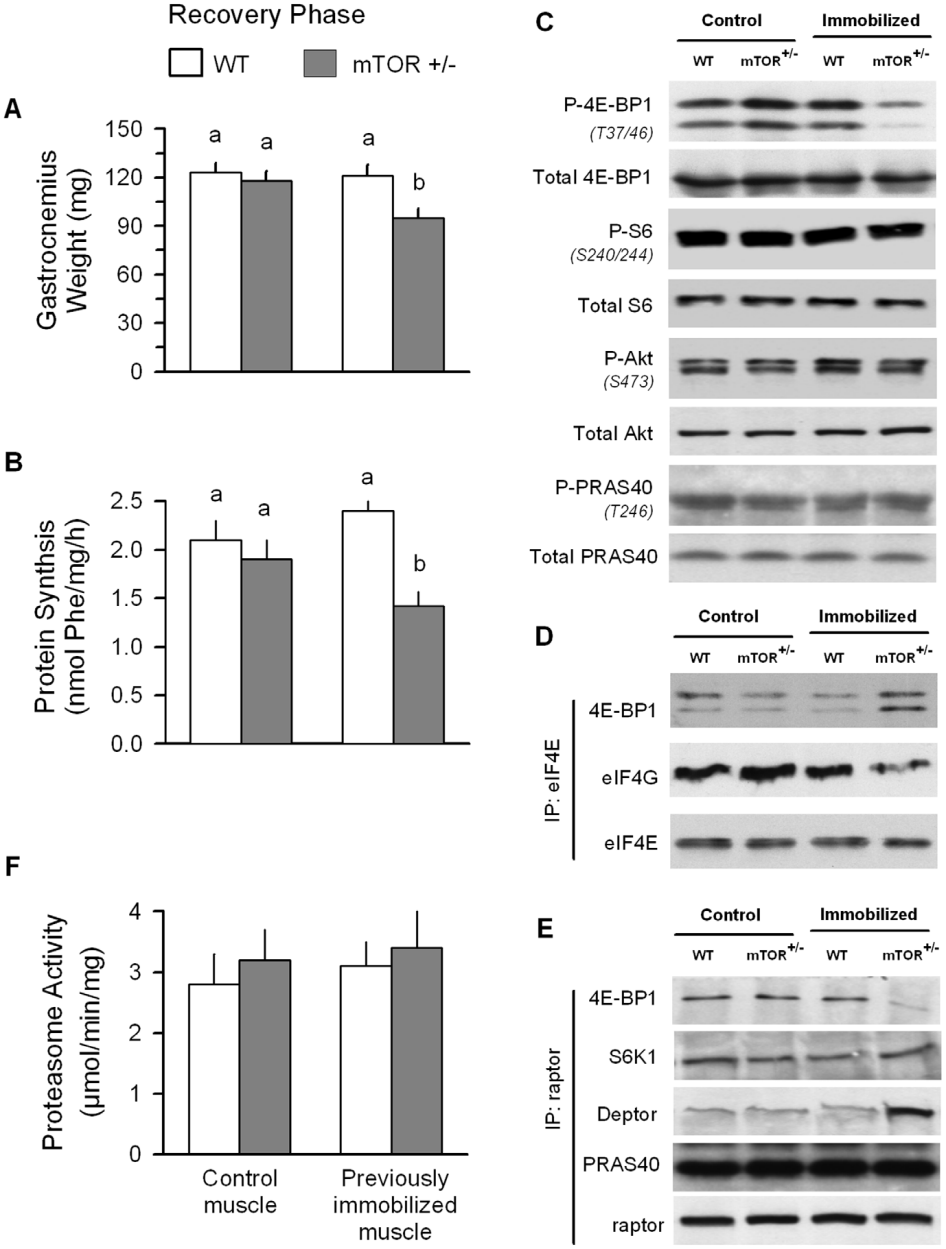
Protein metabolic effects in wild-type (WT) and mTOR^+/−^ mice during recovery from hindlimb immobilization. For all studies, mice had one hindlimb casted for a period of 7 days, the cast removed, and endpoints assessed after 10 days of recovery. (C) Western blot data and are representative of n = 5 per group. (D) eIF4E was immunoprecipitated (IP) from muscle homogenates and immunoblotted for either 4E-BP1, eIF4G or eIF4E, and representative blots are shown. (E) Raptor was immunoprecipitated from muscle homogenates and immunoblotted for either 4E-BP1, S6K1, PRAS40, Deptor or raptor. Data from mice included in this figure are different from that presented in Figure 8. Values with different superscript letters (a,b) are statistically significant (*P*<0.05). For all bar graphs (A, B and F), values are means ± SEM; 7–9 mice per group.

At the end of a 10-day recovery period, the weight of the previously immobilized gastrocnemius from WT mice was not different from the non-casted contralateral muscle (Figure 9A). The rate of protein synthesis (Figure 9B) as well as the phosphorylation of 4E-BP1, S6 (e.g., substrate for S6K1), AKT and PRAS40 (Figure 9C) also did not differ between the two muscles from WT mice at this time. Finally, there was no difference in proteasome activity between control and previously immobilized muscle from WT mice (Figure 9F). In contrast, in mTOR^+/−^ mice the weight of the previously immobilized muscle remained lower than that of the contralateral control muscle at the 10-day time point. This failure to fully recover muscle mass in the mTOR heterozygous mice at this time was associated with a lower rate of protein synthesis, but was independent of a change in proteasome activity. Although no difference was detected between the control and previously immobilized muscle of mTOR^+/−^ mice for phosphorylated S6, Akt or PRAS40, the phosphorylation of 4E-BP1 remained reduced in previously casted muscle, compared to control values from the contralateral muscle.

When the same amount of eIF4E was immunoprecipitated in all groups, the amount of 4E-BP1 bound to eIF4E (e.g., inactive cap-binding complex) was increased in previously immobilized muscle from mTOR^+/−^ mice, compared to either the contralateral control muscle or muscle from the control mice. Conversely, the amount of eIF4G bound to eIF4E (e.g., active complex) was decreased in the reloaded muscle of mTOR^+/−^ mice (Figure 9D).

Finally, raptor functions as a scaffold protein recruiting substrates to mTORC1 [38]. Therefore, raptor was immunoprecipitated and then immunoblotted for 4E-BP1, S6K1, PRAS40 and Deptor. The decreased muscle protein synthesis in the previously immobilized muscle at day 10 of the recovery period was associated with a reduction in raptor•4E-BP1 binding and an increased amount of raptor•Deptor complex (Figure 9E). In contrast, the binding of raptor with S6K1 and PRAS40 was not altered at this time. As Deptor is also a constituent of mTORC2, we attempted to determine the binding of Deptor to this complex, but we were unable to obtain reliable immunoblots of Deptor from rictor immunoprecipitates (data not shown).

### Impact of hindlimb immobilization and reloading on inflammation and IGF-I in WT and mTOR+/− mice

**Figure 10:**
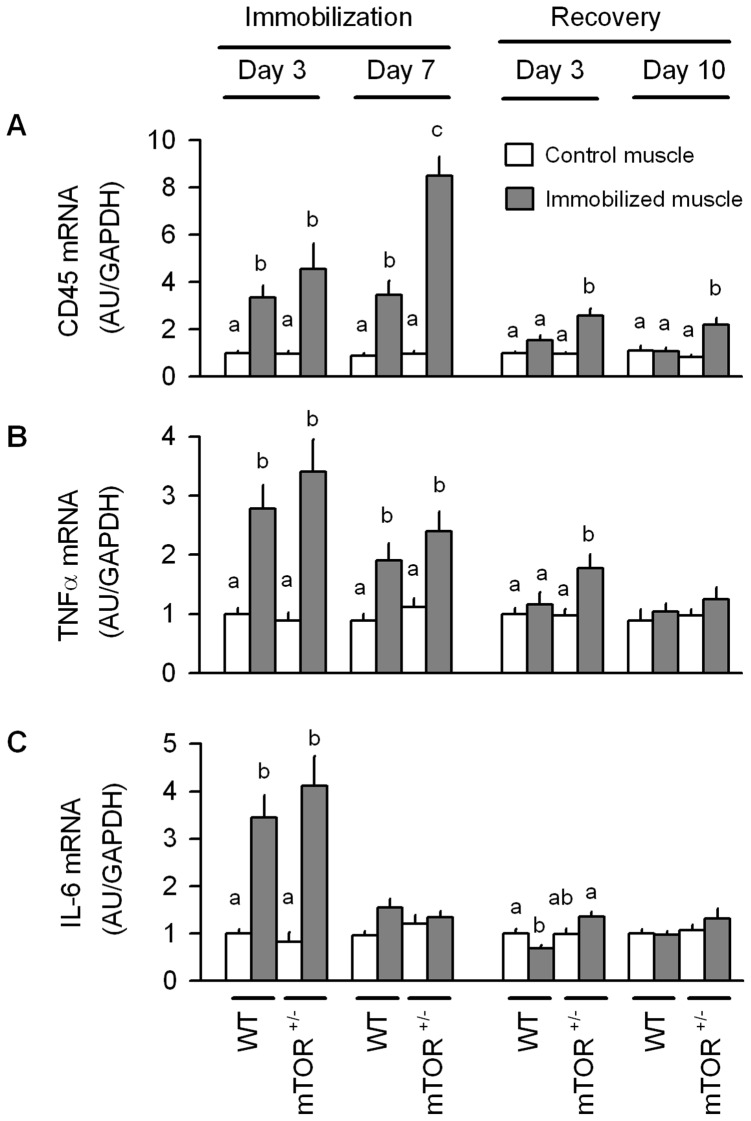
Skeletal muscle mRNA content of inflammatory markers in response to immobilization and recovery in WT and mTOR^+/−^ mice. The relative content of (A) CD45, (B) TNFα, and (C) IL-6 mRNAs as determined by qRT-PCR on gastrocnemius obtained at either 3 or 7 days of unilateral hindlimb immobilization or at 3 or 10 days after recovery from disuse. Data from the immobilized muscle are compared to that of the contralateral non-casted control muscle in the same mouse. All data were normalized to GAPDH and the WT control value was set at 1.0 arbitrary units (AU/GAPDH). Values with different superscript letters (a,b,c) are statistically significant (*P*<0.05). For all bar graphs, values are means ± SEM; 7–9 mice per group.

We also quantified the mRNA content for CD45, a pan-leukocyte marker, as well as the inflammatory cytokines TNFα and IL-6 which have been implicated in mediating the local inflammatory process and the atrophic response [39]–[41]. After 3 days of immobilization, both WT and mTOR^+/−^ mice had comparable increases for muscle CD45, TNFα and IL-6 mRNA (Figure 10A–C, respectively). By day 7 of immobilization in WT mice, CD45 and TNFα remained increased, but IL-6 had returned to values seen in the non-casted contralateral control muscle. A similar response was detected in mTOR^+/−^ mice, except that the immobilization-induced increase in CD45 was greater in the mTOR heterozygous mice than WT animals. The inflammatory state was also profiled during the early (e.g., 3 days) and late (10 days) phase of recovery after cast removal. In WT mice, CD45 and TNFα mRNA did not differ between the previously immobilized and the control muscle after 3 days of recovery; however, IL-6 was reduced by 40%. After 10 days of recovery in WT mice, no immobilization-induced change in local inflammatory markers was detected. For mTOR^+/−^ mice, the increased CD45 and TNFα mRNA seen at day 3 of recovery was greater than in time-matched WT mice, and CD45 was still elevated in mTOR^+/−^ mice after 10 days of reloading.

**Figure 11:**
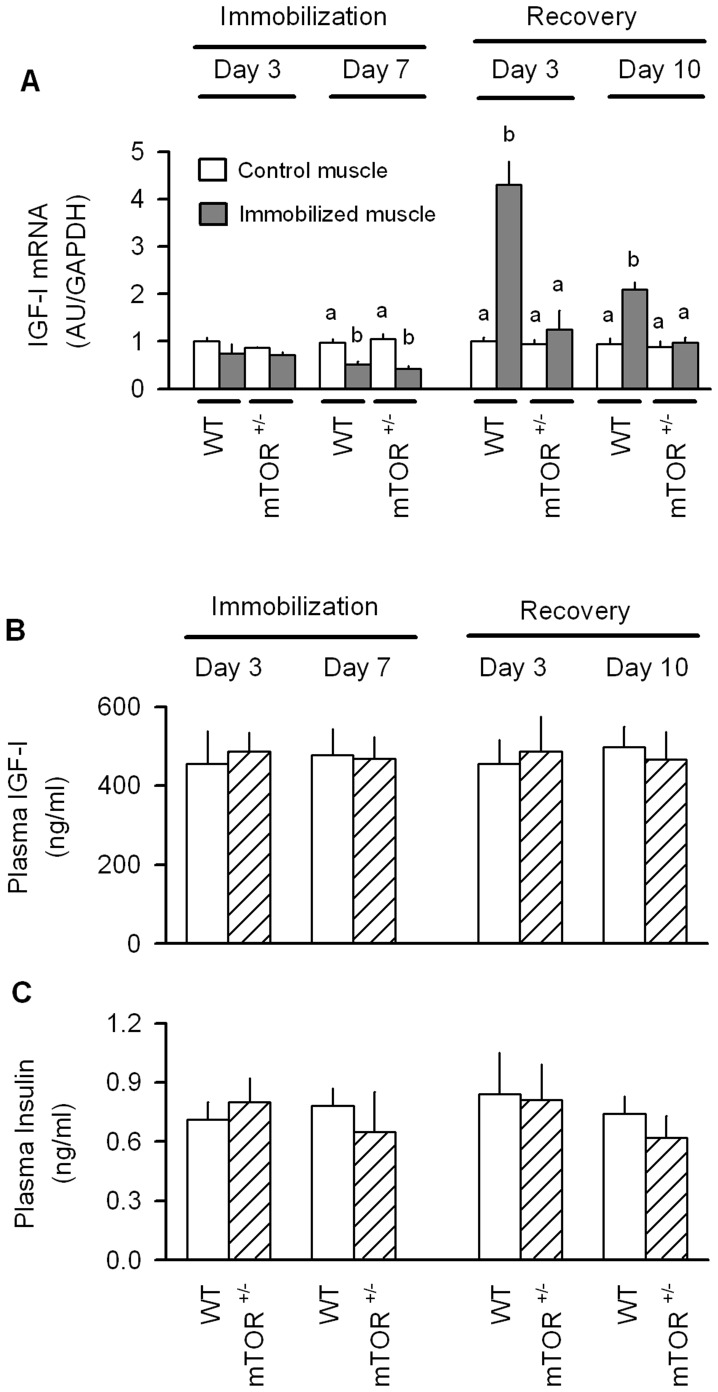
Skeletal muscle mRNA content of IGF-I as well as the plasma concentration of IGF-I and insulin in WT and mTOR^+/−^ mice during immobilization and recovery. (A) The relative content of IGF-I mRNA determined by qRT-PCR on gastrocnemius obtained at either 3 or 7 days of unilateral hindlimb immobilization or at 3 or 10 days after recovery from disuse. The primer probe used recognizes all IGF-I transcripts [80]. Data from the immobilized muscle are compared to that of the contralateral non-casted control muscle in the same mouse. All data were normalized to GAPDH and the WT control value was set at 1.0 arbitrary units (AU/GAPDH). Values with different superscript letters (a,b,c) are statistically significant (*P*<0.05). (B, C) The plasma concentrations for IGF-I and insulin, respectively, did not different between WT and mTOR^+/−^ mice at the time points assessed. For all bar graphs, values are means ± SEM; 7–9 mice per group.

IGF-I is an endocrine and autocrine regulator of muscle mass [42], its relatively importance as a mediator of atrophy and muscle regrowth in various conditions remains controversial [43], [44]. An immobilization-induced decrease in IGF-I mRNA was detected by day 7 of immobilization, but not at the 3-day time point, and this temporal response was similar in both WT and mTOR^+/−^ mice (Figure 11A). An opposite change in IGF-I mRNA was observed in WT mice in response to reloading. That is, the previously immobilized muscle of WT mice exhibited a 4- to 5-fold increase in IGF-I mRNA after 3 days of recovery, compared the non-casted control muscle. IGF-I mRNA was still increased in WT mice after 10 days of recovery. This compensatory increase in IGF-I observed in WT mice during reloading was completely absent in mTOR^+/−^ mice (Figure 11A).

The plasma concentration of IGF-I did not differ between WT and mTOR^+/−^ mice at day 3 or 7 of immobilization or day or 10 of recovery (Figure 11B). Likewise, the plasma insulin concentration did not differ between WT and mTOR^+/−^ mice at any time point assessed (Figure 11C).

## Discussion

Various animal models have been employed to investigate the metabolic changes produced by muscle disuse. The most widely used of these is likely hindlimb suspension, but an atrophic response is seen in response to extended bed rest, denervation, or hindlimb immobilization (either bilateral or unilateral). Each model has specific strengths which encourage its use as well as weaknesses which limit data interpretation [45]. Although other murine models of immobilization have been reported [46], [47], the current series of experiments were performed using a newly developed unilateral hindlimb immobilization model which is easy to implement and requires no specialized equipment, permits comparison of the immobilized to control muscle in the same mouse, maintains neural innervation to the musculature, does not alter food consumption, permits recovery-type studies to be performed, and produces a relatively low level of animal stress. As we are aware of no investigation which reports the temporal progression of changes in both muscle protein synthesis and degradation during both a period of immobilization and recovery in the same animal model, our initial studies focused on the metabolic characterization of this new murine model.

The immobilized hindlimb demonstrated progressive atrophy of the gastrocnemius during the 7 day experimental protocol. It is noteworthy that this localized muscle atrophy did not decrease whole-body lean mass, as assessed by ^1^H-NMR, and this consistent with the unaltered food consumption in casted mice. Disuse atrophy results from an imbalance between rates of protein synthesis and degradation. The consensus from the available literature indicates a reduction in muscle protein synthesis, which commences as early as 6 h after immobilization [11] and remains decreased for several days to weeks [5], [48]. Several previous studies of muscle disuse have revealed a close association between reduced protein synthesis and impaired mTORC1 activity, as evidenced by the coordinate decrease in phosphorylated S6K1 (or S6) and 4E-BP1 [6], [19]–[21], and our current data are consistent with the these previous reports. Additionally, we also detected an immobilization-induced decrease in S473-phosphorylated Akt, similar to that previously reported [18], suggesting either a direct or indirect inhibition of mTORC2 activity. Moreover, immobilization impaired the ability of leucine to acutely stimulate muscle protein synthesis and 4E-BP1 phosphorylation. Such data are consistent with the anabolic resistance seen in human muscle after 14 days of immobilization [14]. Despite the recognized importance of mTOR signaling in regulating protein balance, our data indicate that the immobilization-induced decrement in muscle mass and protein synthesis was comparable in WT and mTOR^+/−^ mice. These data are consistent with the previous work of Bodine et al [18] where in vivo administration of the mTOR inhibitor rapamycin did not alter muscle weight in mature rodents, suggesting that maintenance of muscle weight under basal control conditions is not mTOR-dependent.

The atrophic response to muscle disuse is also partially mediated by increased protein degradation. Although the relative importance of the various proteolytic pathways is still a area of controversy and may appear to be dependent upon the etiology of the atrophic insult [46], however, a diverse array of catabolic conditions activate the ubiquitin proteasome pathway [22], [33]. Muscle disuse increases the mRNA content for the muscle-specific ubiquitin E3 ligases atrogin-1 and MuRF1 [6], [8], [21], [47], [49], [50], whereas knock down of these atrogenes ameliorates the loss of muscle mass induced by denervation or hindlimb suspension [10], [51]. Collectively, atrogin-1 and MuRF1 mRNA expression is routinely used as a surrogate marker for muscle proteolysis. However, while the immobilization-induced up-regulation of atrogenes is commonly detected, the duration of the increase is variable and often transient [8], [47], [50], [52]. In the current study, we found both atrogin-1 and MuRF1 mRNA had returned to levels seen in the control muscle by day 7 of immobilization. However, proteasome activity was still elevated in the immobilized muscle at this specific time point. The discordant association of proteasome activity and atrogene expression may suggest atrogin-1 and/or MuRF1 do not catalyze the rate controlling step in protein degradation. In this regard, cleavage of sarcomeric proteins by calpain may be necessary to provide substrate for the ubiquitin proteasome system [53]. Therefore, it is possible that proteasome activity can be increased without a concomitant elevation in either atrogin-1 or MuRF1. Alternatively, there may be a discordant regulation of atrogene mRNA and protein expression, which was not assessed in the current study because of the relatively limited mass of available muscle. The lysosomal-autophagy pathway also contributes to protein degradation in selected models of muscle atrophy [54]. However, while autophagy-related genes are up-regulated in response to differing atrophic stimuli [54], [55], other studies have reported that such an increase is not evident in immobilized muscle [46]. Finally, the current study did not investigate changes in muscle apoptosis which has been shown to be increased in immobilized muscle [13], [39]. Hence, the relative contribution of autophagy and apoptosis to immobilization-induced in this particular model of disuse remains to be elucidated. Similar to protein synthesis, all measured indices of protein degradation in the immobilized muscle did not differ between WT and mTOR^+/−^ mice during the immobilization phase.

In many chronic catabolic states, elevated blood borne proinflammatory cytokines induce muscle wasting by decreasing protein synthesis and/or increasing protein degradation [53], [56]. Furthermore, myocytes contribute to the innate immune response by synthesizing and secreting a host of proinflammatory cytokines [57], and the local activation of nuclear factor-kappa B is sufficient to produced muscle atrophy [58]–[60]. In this regard, the consistent elevation in CD45 mRNA throughout immobilization suggests a marked infiltration of inflammatory leukocytes. Moreover, the sustained increase in TNFα mRNA and the more transient increase in IL-6, is indicative of a localized muscle inflammation. While we were unable to resolve the temporal progression of infiltrating leukocytes versus cytokine synthesis in our current study, previous work has reported that an in increase in TNFα, IL-1 and IL-6 precede the immobilization-induced increase in CD45 [47]. Based on the sustained increase in TNFα in the immobilized muscle, coupled with the changes in both protein synthesis and proteolysis, we cannot exclude this cytokine as a potential mediator of the atrophic response. The role of mTOR in regulating the innate immune response in leukocytes is poorly defined and may vary depending on whether pro- or anti-inflammatory cytokine production is being examined [61]. In the current study, there was no difference in the immobilization-induced increase in TNFα or IL-6 between WT and mTOR^+/−^ mice. However, mTOR haploinsufficient mice did exhibit an exaggerated increase in CD45 mRNA on day 7 of immobilization, but the cause and physiological importance of this difference remains unclear.

Although TNFα or an unidentified cytokine may directly mediate the atrophic response during the unloading phase, these immunomodulators may also function in an indirect manner by reducing the local concentration of an anabolic mediator. One such potent mitogen is IGF-I which functions both as a traditional endocrine hormone, but also by an autocrine/paracrine mechanism [42], [62]. In this regard, exogenously delivered IGF-I ameliorates wasting produced by excess glucocorticoids [63], denervation [64], and sepsis [65], and the local over-expression of a muscle-restricted IGF-I isoform can prevent decrement in muscle loss seen with aging [66] and neuromuscular disease [67]. In contrast, others have reported that the localized over-expression of IGF-I in muscle does not prevent the casting-induced decrease in muscle mass and force generation [16], [68]. Our data and others [16] show a marked reduction in muscle IGF-I mRNA in immobilized muscle. However, the temporal progression of the immobilization-induced change in IGF-I was not consistent with the accompanying alterations in protein synthesis or atrogene expression. While these data suggest the decreased muscle IGF-I is an unlikely mediator of disuse atrophy, our data should be interpreted cautiously as we assayed all muscle IGF-I transcripts. We did not quantify various splice variants or muscle-specific isoforms of IGF-I which may be differentially regulated and which may have differing bioactivities [69], but whose physiological functions remain controversial [70].

It is important to distinguish between mechanisms responsible for disuse atrophy and those which promote muscle repair upon reloading as they likely differ [71]. Therefore, using the same murine model we also examined the role of mTOR in the recovery of muscle mass. In WT mice protein synthesis is selectively elevated in the previously immobilized muscle within 24 h of reloading and the increase persists for at least 6 days. By day 10 of recovery, both protein synthesis and muscle weight had returned to control values in WT mice. However, despite the normalization of muscle protein synthesis by 10 days of recovery, the previously immobilized muscle still exhibited leucine resistance. Characterization of metabolic changes in both the atrophy and recovery phase is important, as the temporal progression of muscle regrowth appears to be inversely proportional to the duration of immobilization [3], [7], [27], [28], [45], [72]. Moreover, the ability of mice to fully recover muscle mass is in contrast to the more prolonged time for muscle repair and regeneration needed in rats [13], [27], [73].

In contrast to WT mice, there was a significant delay in the reloading-induced accretion of muscle protein in previously immobilized muscle from mTOR^+/−^ mice. The impaired response was caused by a reduced rate of muscle protein synthesis, with no apparent difference in the rate of degradation (e.g., proteasome activity). The decreased rate of protein synthesis in reloaded muscle from mTOR^+/−^ mice was associated with a reduction in phosphorylation of 4E-BP1, but not S6. The reason for this divergent mTOR signaling response is not understood, but emphasizes the need to determine both the activation of specific signal transduction pathways as well as protein synthesis per se.

mTORC1 is a multiprotein complex with raptor functioning as a scaffold protein recruiting a variety of substrates [17]. Raptor binds to the TOR signaling motif found in all known substrates of mTORC1, including 4E-BP1, S6K1 and PRAS40 [74]. In the previously immobilized muscle from mTOR^+/−^ mice which exhibited delay regrowth, the binding of 4E-BP1 to raptor was decreased. This observation is consistent with the endotoxin-induced decrease in muscle protein synthesis and mTORC1 activity previously reported [75] and, conversely, the recruitment of 4E-BP1 to raptor for optimal stimulation of protein synthesis [76]. However, this defect appeared to be relatively selective and was not a generalized phenomenon as the binding of raptor to either S6K1 or PRAS40 was not altered. However, Deptor is a negative-regulator of mTOR kinase activity in skeletal muscle [12] and the interaction of Deptor with raptor was increased in the previously immobilized muscle from mTOR^+/−^ mice. Finally, in contrast to the inhibition of mTORC1 signaling, S473-phosphorylated Akt and T246- phosphorylated PRAS40 did not differ from values from the contralateral control muscle.

The protein metabolic changes in WT mice were temporally associated with an increase in IGF-I mRNA, and the impaired ability of mTOR^+/−^ mice to replete mass was associated with a failure of these animals to increase IGF-I in a compensatory manner. Although somewhat counterintuitive, the up-regulation of the local inflammatory response in muscle is also necessary for efficient muscle recovery from disuse [77], [78]. We did not see a reloading-induced increase in muscle CD45, TNFα or IL-6 in WT mice in the current study and this may be explained by a relatively transient cytokine response which we missed by only sampling tissue at day 3 and 10 of recovery. In contrast, mTOR^+/−^ mice showed a relatively small but sustained increase in CD45 throughout the duration of the 10-day recovery period which was associated with a transient early (day 3) increase in TNFα and IL-6. However, we speculate that the limited difference in the magnitude of the inflammatory response between mTOR^+/−^ and WT mice, is an unlikely mediator for the differences in protein balance and mass during the recovery period. In general, our data are consistent with previous reports concluding that recovery is associated with an increase in the phosphorylation-activation state of various elements of the IGF-I/AKT/mTOR pathway [18], [20], [79] and that locally delivered IGF-I can enhance muscle regeneration during the recovery period [16].

In summary, our data indicate that muscle loss is mediated by rapid and sustained changes in both protein synthesis and degradation which cannot be attributed to a localized change in IGF-I. While these disuse-induced changes are likely mediated through inhibition of the canonical mTOR signaling pathway, a reduction in the total amount of mTOR (as seen in the mTOR^+/−^ mice) in muscle does not exacerbate the metabolic imbalance in the immobilized muscle. However, the ability of mTOR heterozygous mice to increase protein synthesis in response to the anabolic signals generated by reloading was greatly impaired. These data support the contention that the inability to fully activate mTOR in previously immobilized muscle limits muscle regrowth and suggests stimulation of this kinase might be expected to preferentially enhance regrowth, but with little salutary effect on the initial loss of muscle.
